# The Transactions of the Provincial Medical and Surgical Association

**Published:** 1834-07-01

**Authors:** 


*The Transactions of the Provincial Medical and Surgical Association.* Vol. II.—London, 1834. 8vo. pp. 546; coloured Plates.

THE volume before us commences with an account of the first anniversary of the Society whose Transactions it contains. After this follows an address, by Dr. BARLOW, and three essays on Medical Topography. The first is an account of the district of the Land's End, by Dr. FORBES; the second, by Dr. CARRICK and Dr. SYMONDS, is on the Medical Topography of Bristol; and the third is an account of Stourport, in Worcestershire, and its neighbourhood, by Mr. WATSON. It is with considerable reluctance that we pass over these interesting papers without extract or comment, but we must hasten on to matter more immediately practical. Dr. Forbes' essay is especially elaborate, and he promises another chapter, containing the medical history of the inhabitants of the district, on a future occasion.

Dr. BARDSLEY has contributed ***Additional Facts and Observations on the Efficacy of Strychnine in some Forms of Paralysis;*** his former remarks on this subject having been contained in the excellent work entitled " Hospital Facts and Observations," published four years ago. The author seems to us to be one of the few hospital physicians who are conscious of the vantage ground on which they stand, and of the comparative facility with which they might advance the progress of the healing art: had he many rivals, we should not have to regret that the improvement of the practice of physic does not keep pace with that of the auxiliary sciences, pathological anatomy and pharmaceutical chemistry.

After quoting the testimony of Dr. Elliotson and Dr. Graves in favour of the employment of strychnia in some paralytic affections, Dr. Bardsley says,

" My further experience with strychnia leads me to repeat, that it is in such cases of paralysis as seem to arise from diminished nervous excitement, that this remedy is particularly indicated; and, generally speaking, that it is likely to prove more serviceable in paraplegia, unconnected with spinal disease, than in hemiplegia, though I feel confident that it will not unfrequently be found an important remedial agent even in hemiplegic paralysis, when the cerebral mischief is not very extensive, and the nerves are still capable of being acted upon by appropriate excitants. I can state, on the authority of facts occurring within the sphere of my own observation, and that of others, that strychnia has proved curative in cases of palsy of long standing, after the complete failure of the discipline ordinarily adopted in these attacks. The late Dr. Somerville, of Stafford, communicated to me an interesting case of paralysis of the hands, arising from the absorption of lead, in which strychnia removed the disease in less than a month. The patient commenced with the eighth of a grain twice daily, but at length took one grain within the same interval." (P. 202.)

Dr. Bright, too, speaks favourably of it; but observes, that "cases of hemiplegia from the rupture of vessels are not those in which this remedy holds the greatest prospect of success, though, with caution, it may be employed in the advanced stages of convalescence, with safety at least, and sometimes with benefit." Our author also quotes a very remarkable case, given by Dr. Gaskoin, in the Medical Gazette, in which "the patient, a boy twelve years of age, was incapable of moving the head, or any part of the vertebral column, from the place of support." The sulphate of strychnia was given in the dose of $\frac{1}{30}$, which was gradually raised to $\frac{1}{5}$, of a grain three times a day. A cure was effected in six weeks.

A case still more remarkable, if possible, was communicated to Dr. Bardsley in the spring of last year. A young lady, in the twenty-third year of her age, who was not able to walk more than three or four miles without a sensation of weakness and pain in the back, had a severe attack of remittent fever in the autumn of 1826. The dorsal debility increased rapidly, and by the end of May, 1827, she was unable to walk; in the succeeding years she went on from bad to worse, and, by the advice of Sir Astley Cooper and Sir C. M. Clarke, tried the usual remedies of cupping and counter-irritation, without any permanent benefit.

"The following account of her condition, on the 16th July, 1830, was drawn up by her regular medical attendant. 'She has now been ill nearly four years; she has entirely lost the use of her lower extremities, but their sensibility is natural, or rather more acute than usual; there is considerable pain across the loins, extending from one spine of the ilium to the other, and over the whole surface of the sacrum; there is also a diffused tenderness of the integuments of the same parts, and also at times much turgescence and a feeling of fulness; there is considerable weakness of the upper extremities; she sleeps but indifferently in general, in consequence of pain in the joints, particularly the ankles and wrists, which she describes to be a sensation as if the joints were separating by violent extension. The periodical evacuations are regular, and tolerably natural; there is, in the intervals, occasionally some leucorrhœal discharge, which is not continued, but appears in considerable quantity suddenly, and then ceases: it is thin and transparent. The appetite is bad, and almost every article of diet disagrees with the stomach, inducing a sensation of fulness and oppression; the bowels are very irregular, and the evacuations, generally, very slimy and unnatural in their appearance. She is subject to rather frequent attacks of increase of all the dyspeptic symptoms, attended with headach and foul tongue, at which time there is an aggravation of all her painful symptoms. She is just recovered from one of these attacks, and the tongue is now clean and almost natural in appearance; the pulse is eighty-one, weak, and easily accelerated." (P. 208.)

The next day, (July 17th, 1830,) Miss S. began the use of the strychnia, by taking $\frac{1}{12}$ of a grain once a day, and continued to take it in increased doses till the 6th of November. Nothing could be more satisfactory than the effects of the remedy. First, her appetite and digestion improved, and soon after the pain in the back diminished; on the 23d of August she could stand with the aid of her crutches, and on the 5th of October she could walk five or six yards, leaning on her brother's arm only. The improvement continued after the medicine had been left off; and in May, 1831, she was capable of walking slowly for an hour without much fatigue. The largest dose administered was $\frac{1}{3}$ of a grain three times a day.

This case, while it showed the advantage to be derived from the persevering use of this alkaloid, showed also with what extreme caution it must be exhibited; as one third of a grain, taken twice a day, was capable of producing convulsive twitchings, alarming both from their violence and their duration.

Dr. A. T. Thomson, who has used this remedy with advantage in the form of the acetate, supposes that strychnia does not influence the circulation in the brain, even when given in fatal doses. The following case, however, seems to show that this opinion is erroneous.

William Jones, ætat. forty-six, was admitted into the Birmingham Hospital, March 25th, 1831. He was suffering from hemiplegia of the left side of three weeks' standing. On the fifth day after admission he began taking half a grain of strychnia twice a day, and continued to do for five days, without any sensible effect. The dose was then increased to a grain twice a day, which at first produced spasmodic contractions of the muscles; but, as this dose gradually lost its power, and in a few days produced no effect at all, the patient was ordered to take a grain and a half every morning. The first dose, however, produced fatal opisthotonos, the death of the patient taking place three hours and three quarters after the administration of the strychnia.

"*Post-mortem Examination, seven Hours after Death.*—April 16, 1831. External appearances: The fingers were firmly contracted, and the muscular system, generally, was in a state of spastic rigidity.

Brain: A considerable quantity of limpid blood flowed from the division of the scalp; the vessels of the dura mater were also turgid with dark-coloured blood. The arachnoid membrane was opaque and thickened throughout all its inflections, but particularly at the base of the brain. The basilar artery, and the arterial circle of Willis, were somewhat diseased, and spotted with atheromatous deposition. In the right corpus striatum there was an apoplectic clot, dark coloured and grumous, of an irregular figure, and in bulk about the size of a very large walnut. The substance of the brain, in contact and in the vicinity of this clot, which was clearly of previous formation, was in a state of ramollissement in several parts. The lateral ventricles contained about f.℥iss. of yellowish serous effusion. The other parts of the brain were healthy.

"The spine: The investing membranes were found in a highly vascular and injected state; the pia mater was of a florid redness, and everywhere congested with arterial blood; there were four recently extravasated patches of blood between this membrane and the arachnoid, which was also thickened and opaque, as in the brain. These patches were situated opposite the last dorsal and the two first lumbar vertebræ; and in the lowermost a distinct coagulum could be recognised. The spinal marrow itself was healthy. It is also to be regretted that the thorax and abdomen were not examined, owing to the interference of the friends of the deceased." (P. 218.)

Dr. Booth, the narrator of this case, then gives the post-mortem appearances in two dogs killed by strychnia, showing that this poison causes congestion of the brain, contrary to the common opinion, which supposes its action to be confined to the spinal cord.

After giving a number of other successful cases, which we must reluctantly pass over, Dr. Bardsley has some observations on the external use of this powerful alkaloid. He says,

—"I wish particularly to direct the attention of the profession to the external application of strychnia in that obstinate form of paralysis of the levator palpebræ superioris muscle, occasionally the drooping or falling of the upper eyelid, by which the eye becomes totally or partially closed. I have met with three examples of this kind, in which strychnia sprinkled on a blistered surface, established immediately above the orbit of the eye, has been productive of great benefit. It is proper to commence with the application of one sixth of a grain night and morning, progressively increasing the quantity to half a grain or a grain at the same intervals. The employment of galvanism may be advantageously combined with the application of strychnia in these cases." (P. 237.)

We cannot, however, resist the temptation of extracting the table giving the result of the employment of strychnia in paralytic cases, in the Manchester Royal Infirmary, since the year 1830. The internal dose was generally from one sixth of a grain twice a day, to half a grain three times a day. The quantity employed externally was from one fourth of a grain to two grains, sprinkled on the blistered surface twice a day.

In the following tables, I.P., O.P., and H.P., mean respectively in-patient, out-patient, and home-patient.

"*Table of Cases of Hemiplegia treated with Strychnia.*

| No. | Name. | Age. | Duration of Complaint. | Disease. | Result. |
|---|---|---|---|---|---|
| 1 | John Johnson, O.P. | 30 | Two months. | Hemiplegia of left side | Cured. |
| 2 | Mary Harwood, I.P. | 43 | Two years. | Ditto. | No benefit. |
| 3 | John Beech, I.P.* | 25 | Ten months. | Hemiplegia of right side. | Relieved. |
| 4 | Eliz. Worsley, H.P. | 58 | Sixteen mths. | Ditto. | Little benefit. |
| 5 | Thos. Walton, I.P.† | 57 | Two months. | Hemiplegia of left side | Relieved. |
| 6 | Jane Macguire, I.P. | 42 | One month. | Ditto. | Cured. |
| 7 | Owen Connor, I.P. | 33 | Four months. | Ditto. | Relieved. |
| 8 | James Brown, O.P. | 60 | Two years. | Hemiplegia of right side. | No benefit. |
| 9 | Alice Burns, O.P. | 33 | Six months. | Hemiplegia of left side | Greatly relieved. |
| 10 | Mary Kelly, I.P. | 39 | Three months | Ditto. | Cured. |
| 11 | John Royle, H.P. | 61 | Sixteen mths. | Ditto. | No relief. |
| 12 | Charles Astley, O.P. | 29 | One year and a half. | Hemiplegia of right side. | Cured. |

* "Beech persevered in the use of strychnia for more than three months after he was discharged from the hospital, and, when I last saw him, he had almost entirely recovered the power of the right side.

† "Walton died suddenly in the course of two months after quitting the hospital. He had not taken any strychnia during that time. On post-mortem examination the basilar artery was found in an ossified state, and a large clot of blood was detected on the medulla oblongata."

*Table of Cases of Hemiplegia treated with Strychnia.*

| No. | Name. | Age. | Duration of Complaint. | Disease. | Result. |
|---|---|---|---|---|---|
| 13 | Mary Brion, O.P. | 59 | Two years. | Hemiplegia of right side. | Little amendment. |
| 14 | Jos. Medcalf, O.P.* | 33 | Thirteen wks. | Ditto. | Cured. |
| 15 | Susan Scott, I.P.† | 60 | Six months. | Ditto. | Little relief. |
| 16 | James Ingham, H.P. | 39 | Two months. | Ditto. | Cured. |
| 17 | Patrick Dillon, I.P.‡ | 48 | Three years. | Hemiplegia of left side | No benefit. |
| 18 | Mary Furbar, O.P. | 43 | Six weeks. | Hemiplegia of right side. | Cured. |
| 19 | Jonath. Jones, I.P.§ | 60 | Two months. | Ditto. | No benefit. |
| 20 | Cath. Davis, H.P. | 28 | Thirteen wks. | Hemiplegia of left side | Much relieved. |

* "Medcalf continued the use of strychnia for more than thirteen months. During the last three months, the paralytic limbs were galvanised each night and morning.

† "This patient died in a short time after her discharge from the hospital, with symptoms of cholera.

‡ "Dillon was very inattentive to the directions given to him by myself and our intelligent house apothecary, Mr. Lloyd; and I have reason to believe that, on several occasions, he deceived the nurse with respect to taking the strychnia pills.

§ "In Jones's case, many remedies had been previously employed, and with a similar unsatisfactory result."

"*Table showing the Results of the Combined Internal and External Use of Strychnia in twelve Cases of Paraplegia.*

| No. | Name. | Age. | Duration of Complaint. | Disease. | Result. |
|---|---|---|---|---|---|
| 1 | Jane Brown* ......... | 13 | Seven months. | Paraplegia. | Cured. |
| 2 | John Edwards. ....... | 53 | One year. | Ditto, incomplete. | Relieved. |
| 3 | Sarah Jackson ..... | 16 | Six months. | Complete. | Cured. |
| 4 | Thomas Pollitt ..... | 24 | Thirteen mths | Ditto. | Cured. |
| 5 | Isaac Sampson† ...... | 60 | One year. | Ditto. | No benefit. |
| 6 | Timothy Smith ...... | 39 | Two years. | Incomplete. | Greatly relieved. |
| 7 | Mary Walsh ......... | 45 | One year. | Complete. | No benefit. |
| 8 | Joseph Chadwick ... | 36 | Six months. | Ditto. | Cured. |
| 9 | John Christie ......... | 32 | Nine months. | Incomplete. | Relieved, but afterwards became hemiplegic. |
| 10 | John Burgess......... | 35 | One year and a half. | Complete. | Relieved. |
| 11 | Sarah Higgison ...... | 48 | Four years. | Ditto. | No benefit. |
| 12 | John Ellison ......... | 33 | Nine months. | Ditto. | Much relieved. |

* "This girl was brought to the infirmary from a distance of more than sixty miles. She had been under the care of a very eminent surgeon, who had previously tried the usual remedies, but without benefit.

† "This man had led a previous intemperate life, and his constitution was much shattered."

"*Table showing the Result of the External Application of Strychnia in eight Cases of Palsy of the Wrists from Lead.*

| No. | Name. | Age. | Duration of Complaint. | Result. |
|---|---|---|---|---|
| 1 | William Roberts ... | 40 | Three months. | Cured. |
| 3 | Thomas Bennett ... | 36 | Six weeks. | Cured. |
| 3 | James Constantine | 29 | Three weeks. | Cured. |
| 4 | John Logan ....... | 40 | Two months. | Relieved. |
| 5 | William Hays .... | 32 | Six weeks. | Ditto. |
| 6 | Thomas Dane ...... | 54 | Three months. | Ditto. |
| 7 | Charles Wood ...... | 23 | One month. | Cured. |
| 8 | Richard Cranshaw... | 39 | Nine weeks. | Cured. |

"Both Logan and Hays recovered the entire power of the wrists in the course of a fortnight or three weeks after leaving the hospital. I received no further information respecting Dane." (P. 238.)

There is a paper on *Chronic Peritonitis,* by Mr. Edward Thompson. He acknowledges the extreme difficulty of the diagnosis, but says that there is one symptom which he has never known to be absent, viz. "a feeling of uneasiness on descending a stair, or stepping from an elevation." Should this be confirmed by other observers, the diagnosis would be extremely easy; but, until this is the case, we hope that no one will adopt the method of treatment recommended by our author, which is neither more nor less than the regular British panacea, or all-heal—salivation, to wit. Were this paper destined to make an impression on the medical public, we should fear the rise of a peritoneal sect, as there formerly was an hepatic one, by whom patients were "put through long and ruinous courses of mercury, without any benefit." (*Abercrombie on Diseases of the Stomach, &c.* second edit., p. 345). Dii tale avortant omen!

Mr. James Dawson has given *the History of a Case of Lithotomy by the Rectum,* which is sufficiently interesting to induce us to present our readers with a large portion of it.

"Robert Cox, nearly three years and a half old, of diminutive form and sickly aspect, was admitted into the Liverpool Infirmary in January, 1832.

"The history of this child's sufferings, during the past six months, was detailed by his father, and it was sufficiently distressing to lead to the belief of the presence of a stone in the bladder. After a few days of repose the sound was passed, but no calculus could then be detected. The urethra was found to be short and very narrow, admitting, with difficulty, the smallest instrument. The bony outlet of the pelvis was deformed; the space left between the conjoined pubic and ischiadic branches being narrowed to a degree I had never before witnessed; this space, by admeasurement, not exceeding one third of an inch; the course of these branches, to within one quarter of an inch in front of the anus, was nearly parallel; from this point into the tuberosities their divergence was abrupt, leaving ample space for the anus itself." (P. 301.)

After remaining in the hospital a few weeks he was sent home, but returned in five months.

"His mother had entirely neglected him; she had allowed the anus to remain in a state of prolapse nearly all this time, so that the exposed mucous membrane had acquired a deep purple tint, was beset with excoriations and ulcerations, and presented, altogether, a very menacing appearance.

"Some pause was necessary, in order to bring the part into a condition to bear any future exploration.

"Taking advantage of a tranquil state of the rectum, I passed the finger, when its point was abruptly met, about half an inch within (or beyond) the fibres of the sphincter ani, by a bulging downwards, in a pouch-like form, of the upper wall of the rectum, through which membrane could be felt, but not very distinctly, the outline of a solid, and hence, probably, a foreign body, of about the size of a large Spanish olive, which appeared to be firmly seated in its novel situation.

"The bladder was forthwith sounded, when the instrument struck against a stone. The foreign body sustained by, and felt through, the medium of the anterior wall of the rectum, was hence satisfactorily identified with the calculus just detected in the bladder. Here, then, was an instance of sacculated stone. It is useless to inquire whether the pouch was a congenital, or an accidental formation. One thing seemed to be certain, viz. that, owing to the extreme narrowness of the bony outlet, the calculus was inaccessible by the usual lateral operation; indeed, I feared, at one time, it might be so by any other route." (P. 302.)

It was now determined to operate by the rectum, as soon as the state of his health would allow of it. The agony he suffered in passing his urine, though unrelieved by large doses of various narcotics, yielded to the introduction, at stated intervals, of an elastic gum catheter, which had been made to retain its curve without the wire. In order to prepare the rectum for the passage of the urine, astringent and sedative injections were at first employed, but were found to be worse than useless. The injection of cold water twice a day was then tried, with great benefit, for it cured a chronic diarrhœa to which the child had long been subject. The operation was performed in January, 1833, in the following manner.

"He was bound by no ligatures. The nurse supported him on a pillow on her lap, in the usual position. A gum-lancet, having its anterior edge rounded and very keen, was laid flat on the finger, which, thus armed, and oiled, was introduced through the anus, so as to reach a point a short distance beyond the recto-vesical pouch, when its edge was turned upwards, and a decided cut made, by drawing the instrument from behind, forwards, in the median line, through the walls of the pouch, and up to the stone, on the hard surface of which the edge of the lancet was distinctly felt to grate.

"I may mention, that the back part of the blade of the instrument was blunted, which allowed the point of the finger to project beyond it, and which was thus at liberty to direct, as well as to execute, the intended incision. After pausing a few moments, to allow time for the retraction of the divided structures, the finger was again passed, when the calculus was felt to be entangled among a mesh of elastic fibres; hence a second section became necessary, in effecting which the speculum was employed.*

"The calculus was now found seated in the upper, or vesical region of the sac, whence, having been displaced by the finger, it fell into the rectum, from among the valvular folds of which, after eluding the attempt once or twice, it was finally withdrawn by the help of Pellier's double silver wire, which served the purpose of scoop and lever. No blood was lost. The operation lasted nearly five minutes.

"The calculus was found to be much smaller than we had been led to expect. I have omitted to state that, during the operation, a quantity of hardened or dried mucous escaped, along with the fæces, through the sphincter ani; it resembled, in form, the peeled skin of a small apple, and it struck us, at the moment, that this substance might have been coiled round the lower moiety of the concretion, something after the manner of the cup of the acorn, and might, in this way, have added to its apparent magnitude.

"The child passed two liquid stools on the day of the operation, in each of which a clot of blood was found, equal in quantity to about two drachms. He passed a very tranquil night. During the four succeeding days the evacuations from the rectum were chiefly made up of urine. About this period of the after treatment, 'Clot Bey' visited our Infirmary; he expressed great interest about the child, but he gave me no hope whatever of the ultimate closure of the communication between the two outlets. A very slight degree of tenderness was detected on pressure, at the lower part of the abdomen, on the second day after the operation, but this was very promptly and properly treated by the house surgeon, Mr. Simon, and no more was heard of it afterwards. On each succeeding day two liquid motions were passed, the urinous admixture being always perceptible in them; the anus, however, remained free from the slightest appearance of excoriation.

"And now was experienced the benefit of the sphincter ani.

* "The employment of this instrument was productive of great torture to the child; and, as the degree of stretching to which the mucous membrane was exposed by its presence was undesirable in every point of view, it was instantly withdrawn."

The control exercised by the fibres of that muscle kept him clean and dry. It was only when the desire to empty the bowel came on with too much suddenness and urgency to allow time for the bed-pan to be placed under him, that his bedclothes or body-linen were at all stained by the excretions.

"On the tenth day from that of the operation, to my surprise and delight, I found he had passed his urine, to the amount of four ounces, in a full stream through the penis; it was voided without pain, or straining, or spasm. After this, no urine was ever detected in the evacuations.

"The calculus, twenty-four hours after extraction, and spontaneously dried, weighed just sixty grains. A few particles had been separated in the operation. It consisted, externally, of ammoniaco-magnesian-phosphate. The internal part, where seen, consisted of phosphate of lime." (P. 307.)

This poor child was allowed to remain in the Infirmary for six months, and died, a few days after leaving it, of the pestilential cholera.

Mr. GRINDROD has communicated a case of *Hydrophobia.*

"Thomas Pitt, æt. six and a half, was, on the morning of Monday December the 2d, 1833, bitten over the left eye by a rabid dog. The parents of the boy immediately washed the wounds, and took him to a public institution, where, however, no decisive measures were pursued. No further notice was taken of the boy until, upon hearing of the circumstance, I called upon the parents, on the ensuing Wednesday, and, with their consent, excised the parts, and afterwards applied a strong solution of caustic. The boy called at my house frequently afterwards, in order to have the excised parts dressed. They soon healed.

"On the morning of Wednesday, January the 8th, the boy appeared in his usual health playing with his associates. About two o'clock in the afternoon he attempted to drink some water, but, on elevating the vessel to his mouth, he trembled, and declared he could not drink any. This circumstance did not excite the suspicion of his parents, who attributed it to the effects of the cold water on the teeth." (P. 311.)

The nature of the disease soon became manifest, and the child died early in the morning of January 11th. The following were the more remarkable post-mortem appearances:

"The mucous membrane of the trachea, immediately before its bifurcation, was highly vascular. The submaxillary and sublingual glands were much enlarged.

"On opening the pharynx, it was evident that this part had been much affected during life. The greater portion of its inner surface was intensely inflamed, and terminated in a defined line opposite the cricoid cartilages. The velum pendulum palati, uvula, and most parts of the pharynx, shewed considerable development of the mucous glands. The tonsils were slightly enlarged. The œsophagus was greatly inflamed near its termination, and in one part the mucous membrane was slightly abraded. The whole mucous membrane was so softened, that the slightest touch of the scalpel separated it.

" *Abdomen.* The stomach was remarkably contracted and completely empty. Intense inflammatory patches were seen on the rugæ, which were very much developed. The mucus was very thick, and had, in some parts, an unusually deep colour. On one of the rugæ was observed a dark spot, about the size of a small shot. The stomach appeared healthy between the rugæ, but had, in some places, a darker colour than natural. The mucous membrane of the duodenum displayed intense inflammation also, and the glandulæ solitariæ were exceedingly enlarged, and very numerous." (P. 325.)

The bitch was dissected by Mr. Jordan, who found the following appearances:

"The lungs were of a very rosy colour, but their structure was healthy. The quantity of mucus in the bronchial tubes was small. The mouth healthy but very parched. The whole of the pharynx was of a deep rosy colour, which terminated in a defined line immediately opposite the cricoid cartilages. The larynx was healthy. A considerable vascularity was manifest at the lower part of the œsophagus, and was continued into the stomach. On opening the stomach, a mass of substance was observed, which had certainly the external appearance of a bird's nest. On being separated, it was found to consist of straw, wood, leather, &c. These materials were in a remarkably dry state. Upon being removed, the mucous membrane was found to be covered, in many parts, with a tough mucus. The rugæ were particularly manifest, and upon their surface were observed, here and there, intense inflammatory patches, and, in other parts, black or dark brown streaks, which, in a few hours, became still more perceptible. The external surface of the stomach seemed to be healthy. The par vagum and nerves of the tongue were perfectly healthy. The salivary glands were of the usual colour and size." (P. 326.)

Mr. Prichard relates a very remarkable case. A lady, aged sixty-eight, who was supposed to be suffering from renal calculus, passed a large biliary concretion by stool, in November, 1828. She had previously been under our author's care, and again sought his advice in May, 1829. From this time till her death, in September, 1830, she laboured under so perplexing a multitude of symptoms, that the diagnosis was difficult, if not impossible. Under the date of February 16th, 1830, we find it stated, that, " on examining the back, a tumor was discovered in the region of both kidneys; that on the right side being circumscribed, and much more elevated than that on the left: on both sides, but more especially on the right, a pulsation, synchronous with that of the radial artery, was clearly perceptible, and I find in my note the following question, 'Aneurism, or abscess with pulsation communicated?' Whilst these tumors, especially that on the right side, went on increasing, the power of moving the limbs daily improved, and the general health remained stationary." (P. 344.)

Large purulent discharges from the bladder afterwards occurred, with temporary relief: on one day two ounces of pure pus were passed. The following was the solution of some of the difficulties.

" *Post-mortem Examination, twelve Hours after Death.* The general appearance of the abdominal viscera, *in situ*, natural.

" The stomach contained a quantity of dark fluid, by which it was much distended.

" The spleen was very small.

"The liver was unnaturally small and very pale, except in the neighbourhood of its anterior edge on either side of the suspensory ligament, where it was pretty much coloured by bile. On a cursory examination, it was difficult to discern any thing like a gall bladder, but, on further investigation, this organ became evident, in the form of a semi-cartilaginous membrane, puckered up into several elevated folds, within one of which a small gallstone was lodged. No bile, nor appearance of bile, could be detected in any part of this membrane. The cystic duct, of a natural size, proceeded downwards, from a portion of it, and its internal coat was deeply tinged with bile. The hepatic duct was natural, as were the pori biliarii and the ductus choledochus. The right kidney, much enlarged, weighing twelve ounces, formed the external tumor. Its adipose and cellular investment adhered firmly to it, and the lower and back part of which shewed marks of recent inflammation, being, in its thickened state, inseparable from the organ itself. The renal arteries, veins and ureter, were healthy, excepting that the latter contained curdy pus. A section of this kidney exposed a mass of extensive disease, the centre of which had a firmly gelatinous character, and was intersected by white bands. In other parts the appearance was tubercular, more or less advanced to a state of ramollissement; its superior portion, from which the discharges of curdy purulent matter from the bladder proceeded, was in a state of suppuration.

" The left kidney weighed four ounces, and was tolerably free from disease, excepting that, on its surface, a small hydatid had formed.

" The bladder, small and very much thickened, contained about ℥iss. of thick fœtid urine, mixed with the curdy pus from the right kidney.

"The aorta, where it passed over a considerable protuberance of the spine, firmly adhered thereto, and its external coat was inflamed in patches, and, in parts, firmly ossified.

"A considerable protuberance presented itself about the last dorsal and first lumbar vertebræ, and the psoas muscles, on either side, bulged out laterally. On removing this portion of the spine, and examining it, it appeared that the spinous processes of the last dorsal, and the first and second lumbar vertebræ, were separated from their bodies, and partook of the morbid changes surrounding them. The first lumbar vertebra was converted into a soft carious mass, having partially the same character of tubercular ramollissement as the kidney. The dura mater, in the vicinity of this mass of disease, appeared healthy; on opening it, the medulla spinalis had evident marks of recent inflammation, and the pia mater investing it and the proceeding nerves were much injected. On removing the psoas muscles, several tumors, as large as a walnut, appeared, closely attached to the spine, two on one side and one on the other, having the same character of scrofulous tubercle as the other diseased parts." (P. 345.)

How and when the gallstone passed into the bowels was a question not resolved by the dissection, and must be considered a curious problem in pathology.

Mr. Watson gives an account of a case of *Uterine Hydatids.* The patient was twenty-two years old, and had been married ten months. There were several discharges of hydatids, followed by a flow of blood from the uterus, which, after resisting medicine, yielded to injections of port wine and water. The patient's recovery was perfect.

We are indebted to Mr. Thomas Davis for the account of a case which is probably without a parallel. A boy accidentally shot himself, driving a piece of stick three inches in length between the third and fourth ribs on the right side, where it disappeared. He survived the wound thirty-seven days, and, on examination, the stick was found in the right ventricle of the heart, incrusted with a thick coagulum as large as a walnut. There was neither wound nor cicatrix, either in the heart or pericardium. Mr. Davis supposes that "the stick, after wounding the lung, passed into the vena cava, and was carried by the stream of blood, first into the right auricle, and then into the right ventricle, where it became fixed."

Mr. W. F. Morgan, late house-surgeon to the Bristol Infirmary, gives the method of *reducing Dislocation of the Shoulder* practised in that institution. It invariably succeeds in recent cases, of which Mr. Morgan saw at least 150, as pupil and house-surgeon. The following is the method recommended.

"As soon as the patient is seen, and the nature of his injury ascertained, he is placed sideways on a common chair, with the dislocated arm hanging over its back, which is padded for the reception and support of the axilla. A reel-towel is then fastened on the arm, immediately above the condyles, by a running noose, with the knot outwards; and its lower part is formed into a loop some way above the ground, to receive the foot of the surgeon. One or two assistants press on the shoulder, much in the same way as Mr. Toogood advises, to fix the scapula and prevent the patient from rising, and the surgeon puts his foot, or, occasionally, his knee, into the loop of the towel, and gradually and unremittingly bears on it with the whole weight of his body. In a very little time, generally within three or four minutes, the head of the bone slips into its place with an audible jerk; the patient's sufferings are moderate, and of short duration, and the use of his arm is soon restored." (P. 362.)

Dr. Ward has communicated *Observations upon Cholera, as it appeared in Wolverhampton and its Neighbourhood, in August, September, and October,* 1832. This is a sensible paper, and evidently written by a practical man, but does not contain much matter that would be new to our readers. Dr. Ward speaks highly of Dr. Stevens's method, but thinks croton oil the best remedy in the stage of collapse.

In the *Observations on the Peculiarities of the Diseases of Infants and Children,* by Dr. J. K. Walker, we find the following passage:

"There is reason to believe that the uvula may, from various causes, be in a state of hypertrophy and relaxation, so great, as to keep up a constant irritation, and, in the opinion of Baron Larrey, even to occasion actual suffocation. I have seen, in many instances of laryngeal affections in adults, the most decided relief follow the removal of a portion of the uvula, which, by its elongation, was a source of continued irritation. In one such patient, I had the pleasure to find an almost immediate amendment in the cough, as well as other symptoms; and, no doubt, cases occur, where this state of the uvula has often been overlooked, and the cough and other symptoms have been attributed to a chronic disease of the larynx. It will sometimes happen that, in epidemic fever, accompanied with sore throat, the uvula is the first part affected, projecting into the rima glottidis, and threatening instant suffocation, In such cases the breathing is embarrassed, the voice extinguished, and the deglutition impeded. Yet, have not such symptoms occasionally occurred, without the real source of the mischief being even suspected? A case of this kind is recorded by Baron Larrey, where excision of the uvula gave instant relief. The patient was threatened with immediate suffocation, and, on inspection of the throat, the uvula was found drawn down into the rima glottidis, to the extent of nearly half an inch, by the act of inspiration. By its removal, perfect freedom was given to the respiration, and the patient was extricated from impending asphyxia." (P. 408.)

There is nothing so new, say the French, as that which has been forgotten. Many of the unpleasant symptoms arising from affections of the uvula have been described by that vigorous but somewhat affected writer, Aretæus: in his chapter Περι των κατα κιονιδα παθων, he says, for example, Πνιξ δε επ' αυτεοισι πασι, και ἥκιϛα ρηιδιως καταπινȣσι· Βηξ επι πασι μεν, μαλιϛα δε επι τοισι Ιμαντιοισι, και επι τοισι Κρασπεδοισι. γαργαλισμος γαρ της αρτηριης απο των ὑμενων γιγνεται· εσθ' ὁπη δε και ενϛαζει τι τον ὑγρȣ λαθρη ες την αρτηριην, ὁθεν αναβησσȣσι. Επι δε τῃ Σταφυλῃ, και τῳ Κιονι, δυσπνοια ετι μαλλον, και καϱτα πονηρη καταποσις. κ. τ. λ. (P. 8. Ed. Wigan.)—*i. e.* in all these affections there is a sense of suffocation, and the patients swallow with great difficulty; in all there is a cough, but particularly in those called *Himantion* (strap) and *Kraspedon* (fringe): for the trachea is irritated by the membranes, and in some cases moisture drops into the trachea, and causes coughing. But, in the affections called *Staphyle* (grape) and *Kion* (uvula), the dyspnœa is still greater, &c.

Dr. Walker gives three interesting cases, in one of which enlargement of the liver, and in the others obscure abdominal diseases, were cured by the hydriodate of potass. The ages of the patients were eleven, three, and two and a half.

MR. E. A. JENNINGS has contributed *an Attempt to ascertain, by Experiment, the exact Differences between the Changes produced in the Lungs of Stillborn Children by their Artificial Inflation, and those produced in the Lungs of Stillborn Children by Natural Respiration.*

Our readers are well aware of the test proposed by M. Beclard, who asserts that, when the lungs of a stillborn child float, in consequence of artificial inflation, the air may be squeezed out of them, and they will then sink. Our author gives seven experiments, all of which go to confirm the truth of this ingenious test. When the child had been stillborn, and the lungs artificially inflated, the lungs could always be made to sink, by compressing them. When the child had breathed, it was impossible to effect this, without completely mashing the lungs. In one very curious case, where the child had breathed imperfectly for half an hour only, the right lung floated, and the left lung sank, with the exception of a small part about its root.

There is another test, proposed by Professor Brent, and quoted by our author. It is as follows:

"'In the fœtus, the arterial duct proceeds from that part of the trunk of the pulmonary artery where it divides into two great branches, and running parallel with the arch of the aorta, and in contact with it, joins it at a very acute angle. If the child has breathed, even for a few moments only, the aperture by which the duct enters the aorta becomes oval; and, afterwards, the diameter gradually, but rapidly contracts at the aortal end, so that the vessel forms a cone, with its base towards the pulmonary artery. If the child has breathed several hours, or days, it resumes its cylindrical form, but becomes all much contracted and shorter; so that, from being equal in diameter to the pulmonary trunk, it becomes intermediate between that and the pulmonary branches, or, even, not larger than the latter. If the child has lived above a week, it is wrinkled, and no thicker than a crowquill, while the pulmonary branches are, at least, as large, and the pulmonary trunk as thick as a goosequill." (P. 438.)

On this point the author says,

" Before respiration the arterial duct is of equal diameter throughout its course, considerably larger than either of the pulmonary branches, and nearly as large as the pulmonary trunk.

" As far as these experiments go, they fully confirm the above statement, which corresponds with what Professor Brent states upon the subject.

" 6th. After respiration has been established, the arterial duct becomes conical, the apex of the cone being towards the aorta; it is also considerably smaller in size than the pulmonary trunk, and one or both of the pulmonary branches will be found, in diameter, nearly equal to, if not exceeding it.

" In experiment 7, where respiration had evidently existed in one lung only, it will be observed that one pulmonary branch had increased in size, while the other remained much smaller than the ductus arteriosus. Imperfect as the respiration was in this case, yet the conical form of the duct, and the right pulmonary branch being as large as the duct, showed the changes effected by respiration in these parts with sufficient clearness to leave no doubt on the subject of its having taken place. My observations do not accord with Professor Brent's on the appearance of the orifice at the termination of the ductus arteriosus in the aorta. He states that if a child have breathed but for a few moments, the aperture becomes oval. I have not been able to satisfy myself on this point. In experiments 1, 2, and 3, when respiration had not taken place, it was impossible to say whether the aperture were round or oval; and in cases where it had existed, I have found the same difficulty in deciding on its form. Until respiration has existed for some time, when the aperture becomes puckered, (as in experiments 5 and 6,) I confess my inability to decide on the form of the opening, from the facility with which it may be made to assume either a circular or oval shape." (P. 454.).

We are surprised that he should say, "that from the moment that respiration commences all the blood of the body circulates through the lungs," (p. 452;) for the foramen ovale is usually open for some days after birth, and it is reasonable to suppose that blood passes through it.

Mr. Jenning's paper deserves attention, and we trust that his experiments will be repeated by other physiologists.

The *Reports of the Birmingham Infirmary*, and *Eye Infirmary*, are well drawn up; but the length to which this article has already extended will prevent our giving any account of them.

The volume concludes with *a Memoir of Dr. Darwall,* equally remarkable for its clear and graceful style, its sound common sense, and the lofty generosity of its sentiments. We are indebted for this specimen of what biography should be to that accomplished physician, Dr. Conolly.

We recommend our readers to add this volume to their libraries: some of the papers are immediately and practically useful, and others will supply the philosophic practitioner with ample materials for thinking.

